# The effect of an Empowerment-Based Human Centered Educational Program on early weaning

**DOI:** 10.1017/S1463423621000013

**Published:** 2021-08-25

**Authors:** Naciye Irmak, Arzu Uzuner, Saliha Serap Çifçili, Sevim Aksoy, Burcu Başaran, Merve Tellioğlu

**Affiliations:** Family Medicine Department, Marmara University School of Medicine, Pendik, İstanbul

**Keywords:** breast milk, breastfeeding, education

## Abstract

**Aim:**

To determine the effects of an Empowerment-Based Human Centered Educational Program on early weaning.

**Background:**

Exclusive breastfeeding (EBF) means that the infant receives only breast milk during the first six months of life. It is essential for the healthy growth of the infants and is supported by the main health organizations all around the world. Intervention studies performed within the antenatal period suggest that the education extends the duration of breastfeeding and increases the frequency of EBF.

**Methods:**

This is a semi-randomized control study. An interactive training module including role-plays which contain traditional patterns, short films, short presentations, and group practice with models was prepared. The participants were recruited in the study based on the voluntary participation of pregnant women followed up for antenatal care in a total of four family health centers in Pendik, a district of Istanbul. The pregnant women of one center formed the control group while others constituted the intervention group. All of them were certified as “Baby Friendly” institution. A pretest and a posttest have been performed to measure breastfeeding knowledge in the intervention group. The mothers of the intervention group have been randomized into two subgroups: one group for reminder call and the other for routine follow-up. All the participants including control group were called at the end of the postpartum sixth month to determine the rates of EBF.

**Results:**

Statistically significant increase in the rates of EBF at the end of six months showed the effectiveness of the education module (42.9 versus 22.2%, *P* = 0.001). Supplementary food taking time was earlier in the control group (18.5 ± 8 versus 15 ± 9.5 week, *P* = 0.03). The main reason of early weaning reported by the mothers was “the insufficiency of the mother’s milk.”

**Conclusion:**

The group training and postnatal reminders were found to be more effective than the individual training provided at the baby-friendly health institutions in terms of the effectiveness on increasing the frequency of EBF.

## Introduction

World Health Organization (WHO), United Nations Children’s Fund, American Academy of Pediatrics, and American Academy of Family Medicine recommended that infants should be breastfed exclusively during the first six months of life, supplementary feeding should be started at the seventh month, and that breastfeeding should continue until the baby reaches two years of age (Stuebe *et al*., 2006; World Health Organization, 2014).

Breastfeeding is a natural way to feed a baby. However, most mothers have difficulties at the beginning and they need counseling and family support for a successful breastfeeding. Therefore, in order to increase breastfeeding rates, all health workers should inform mothers adequately about correct breastfeeding techniques during the antenatal and postnatal visits and should profit of any opportunity for patient education (Gartner *et al*., 2005).

Several studies show that mother’s breastfeeding decisions are made during consultation with doctors and nurses during antenatal controls, and support provided by professionals increases the frequency of EBF (Bertini *et al*., 2003; Forster *et al*., 2003; Sheehan *et al*., 2003; American Academy of Pediatrics, 2012). The intervention studies performed within the antenatal period suggest that the education prolongs the duration of breastfeeding (Lumbiganon *et al*., 2012). Group training, especially structured for teaching breastfeeding during the antenatal visits, is found to be more effective than individual antenatal visits. Rosen *et al*. (2008) and Imdad *et al*. (2011) have reported that breastfeeding counseling provided during the antenatal period has a positive effect on breastfeeding only for four to six weeks postpartum; however, if the counseling is integrated to the postpartum visits, the counseling will be much more effective in extending the breastfeeding duration to the sixth month (Imdad *et al*., 2011). Since 1991, Turkish Republic Ministry of Health (T.R.MoH) and UNICEF have cooperated in the “Breastfeeding and Baby-Friendly Health Institutions Program” in our country. However, in Turkey Demographic and Health Survey (TDHS) 2018 report it was indicated that breastfeeding frequency did not reach the desired level. According to this report, the rate of EBF infants during the first month of life in Turkey was 59.2%, this rate decreased to 45.1% at the third month and to 14.4% between the fourth and fifth months (Turkey Demographic and Health Survey, 2018).

In this study, it was aimed to determine the effect of an “*Empowerment Based Human Centered Training Module*” which starts during the last trimester of pregnancy and continues in postpartum period, on the early start of early weaning.

## Materials and methods

### Study design

This study was designed as a semi-randomized controlled study.

### Location of the study

A total of 132 pregnant women who were followed up for antenatal care in a total of four family health centers (FHC) in Pendik, a district of Istanbul, have been enrolled in the study based on their voluntary participation. Pregnant women from one center formed the control group while others formed the intervention group. FHC close to the University hospital were selected as the intervention group so that the pregnant women could easily participate in the training sessions. The control group was selected from a FHC which was far from the intervention FHC in order not to affect the control group. All of the FHC were certified as “Baby Friendly” by the Ministry of Health, in the context of the “Breastfeeding and Baby-Friendly Health Institutions Program.” As stated in the “Prenatal Care Management Guide” published by T.R.MoH in 2014, breastfeeding counseling is recommended to be provided during the antenatal visits in the second and third trimesters of the pregnancy (Turkish Republic Ministry of Health, 2014). These recommendations are best applied in FHC where the health personnel are trained and certified for this purpose. So that, these selected centers are accepted to provide a standard breastfeeding counseling routinely and to promote EBF.

### Sample size

The sample size was calculated using CDC-Epi-info^TM^ calculator program for randomized controlled studies. Forty-one participants for each of the intervention and control groups; the sample size was calculated for a confidence level of 95%, 90% power, expectation for the difference to be three times, between EBF rates before (23%) and after (60%) (Onbaşı *et al*., 2011).

### Participants and study’s flow charts

One hundred and thirty-two participants were randomly assigned into 2 groups (82 participants in intervention group and 50 participants in the control group). Then, 82 participants were assigned into 2 subgroups.

**Figure 1.**
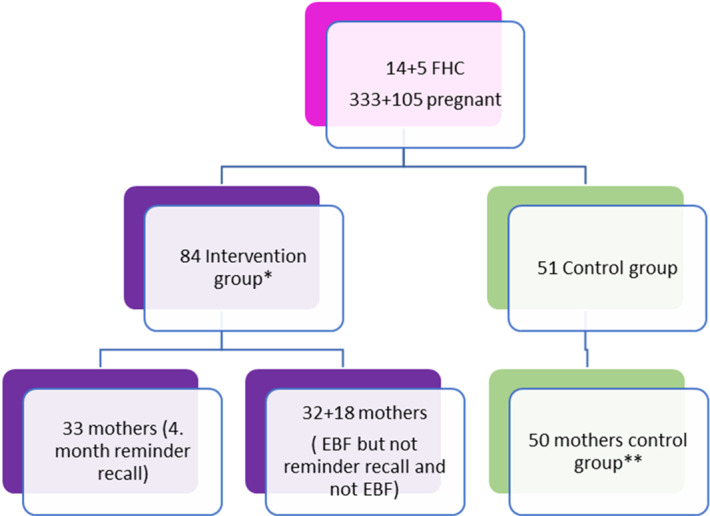
A flow chart of the patient enrollment. (*2 pregnant have early trimester; **1 baby died)

First of all, an informed consent form was obtained from all mothers who participated in the study (Figure 1).

#### Intervention group

A total of 333 pregnant women, registered in the list of 14 doctors in 3 intervention FHC, who were in the second (14–26 weeks) or in the third (27–40 weeks) trimester of their pregnancy were invited by phone to participate in the study. A total of 82 pregnant women were enrolled.

#### Control group

A total of 105 pregnant women (second and third trimesters) were registered into 5 family doctors patient lists who work in the control FHC. These patients have been informed about the study and have been invited to participate in; 50 women accepted and fulfilled face to face the questionnaire at the beginning of the study. The women were called by phone according to their estimated date of delivery, and the actual date of birth of the babies was noted as learned from the mothers; the breastfeeding period of the babies has been evaluated at the sixth month after delivery.

### Training and follow-up

Pregnant women in the intervention group who agreed to participate in the study were invited to the hospital for the first training session. After the training, the participants continued their routine pregnancy follow-ups.

#### Postpartum 7–14 days

The researchers called the participants of the intervention group by phone in their first two weeks after delivery based on their expected birth date. They inquired the mothers about any inconvenience due to breastfeeding and invited them to the hospital with their babies for well-baby visit in their second month after delivery.

#### Second-month follow-up of the infants

During this examination, the babies were routinely examined and their weights were measured and recorded, and percentiles were calculated. The baby’s growth curve was drawn and the chart was given to the mothers. The adequacy of breastfeeding was assessed and mothers were informed.

#### Randomization at the fourth month

Based on the literature, the fourth month seems to be a breaking point for early weaning. To investigate the effectiveness of intervention at the fourth month, participants were randomized into two groups following the fourth month visit by phone: intervention group 1 and intervention group 2. The 32 patients in group 1 were recalled at the fourth month and 33 patients in group 2 were not recalled until the end of the study. It was determined that 17 babies had been started already early weaning food at the second month visit.

#### Last visit by phone at the sixth month

All mothers who were in intervention and control group were called by phone and asked to fulfill the second questionnaire.

### Breastfeeding training during pregnancy

*“An Empowerment Based Human Centered Training Module”* was developed by the authors. Its content is summarized in Table 1. This training has been prepared by taking into consideration the principles of adult education, and the most appropriate breast milk trainings in the literature were searched.

**Table 1.** An Empowerment-Based Human Centered Training Module

	Total	(1 h 45 min)	105′
Module	Title	Issue	Time (min)
1.	Opening	The purpose of the meeting	5′
2.	Acquaintance	The participants identified themselves	10′
3.	Role-play	2 cases – grand group discussion	30′
4.	Coffee break		10′
5.	Slideshow	The importance of breastfeeding	20′
6.	Short film	The demonstration of breastfeeding	5′
7.	Practice	The technique of breastfeeding	10′
8.	Evaluation	Summarize of what we learned – get feedback	15′
	Total	(1 h 45 min)	105′

Adults prefer learning based on practical problem solving, in small groups of 6–8 persons so that they could benefit from the experience of the others. Adults learn better when they are actively involved in the session, so that interactive teaching/learning methods must be used. The use of the models and the maquettes are tools to facilitate practical learning. Adults’ behavioral change is affected by their past experience and the environmental factors such as physical comfort and pleasant surroundings; a dynamic interactive program is essential so that the adults feel free to express their ideas, their views, and their feelings. The participants also must be volunteer for education; they like to get rewards such as certificates to encourage their learning process. (Nadler, 1984; Merriam, 2001; Sürücü, 2014).

The training in this study was planned for 6–8 participants and baby models were used to teach mothers how to carry the baby and how to breastfeed them. An interactive training module including role-plays, short films, short presentations, and group practices with models was prepared. In addition, presentations were made on information, attitude, and behavior change as stated in Çifçili *et al*.’s (2011) study about barriers against EBF. The traditional patterns were imbedded into the role-play. At the end of the program, they were offered a brochure and some supplies for baby care, as a gift.

The participants were asked to fulfill a questionnaire that measured their knowledge about breastfeeding before the training. At the end of the training, the same questionnaire was asked to be refilled to measure the effectiveness of the training module. And, a “Training Evaluation form” was asked to be fulfilled at the end of session, as a feedback to the trainers.

### Statistical analysis

Statistical analysis was performed in SPSS program. Besides the frequency distribution of the variables, cross analysis has been executed to determine the effectiveness of the intervention. Student’s independent (unpaired *t*-test) test was used for variables with normal distribution and Mann–Whitney *U* test for non-normal distribution. *P* < 0.05 value was considered statistically significant.

## Results

In the intervention group eighty-two mothers with their babies and in the control group 50 mothers with their babies; as two mothers had twins a total of 52 babies formed the participants of the study. The sociodemographic characteristics of the participants are shown in Table 2.

**Table 2.** Sociodemographic characteristics of the participants

		Intervention group	Control group	
		Mean ± SD	Mean ± SD	*P*
Age (year) (*n* = 130)		28.9 ± 5.0	28.9 ± 5.8	0.97*
Educational status (year) (*n* = 125)		8.3 ± 4.2	7.6 ± 3.5	0.45**
		Median	Median	
Monthly income (Turkish Liras) (*n* = 98)		1850	1500	0.57**
		*N* (%)	*N* (%)	
Working (*n* = 132)	Yes	17 (79.3)	6 (26.1)	0.2***
	No	65 (59.6)	44 (40.4)	
Housewife (*n* = 132)	Yes	65 (60.7)	42(39.3)	0.5***
	No	17 (68)	8 (32)	

*Independent sample *t*-test; **Mann–Whitney *U* test group statistics; ***Chi-square test.

**Figure 2.**
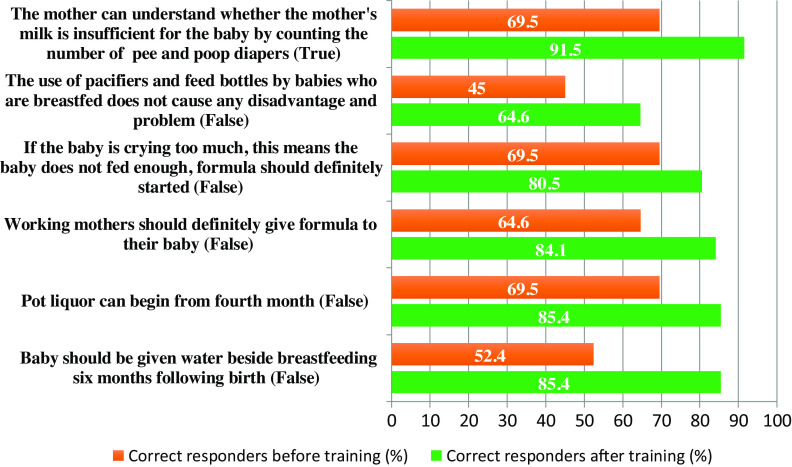
Answers of participants before and after training.

A questionnaire about breastfeeding and the supplementary food is filled before and after the training by the intervention group to evaluate the effectiveness of the training. The answers are summarized in Figure 2.

In the intervention group, pre-training questionnaire total score was (mean ± SD) 11.1 ± 2.0, and the post-training total score was (mean ± SD) 12.7 ± 1.2. These results significantly showed the effectiveness of the training module (*P* < 0.001) on the duration of EBF.

### The frequency of EBF for the first six months

The frequencies of EBF for the first six months after delivery in both groups are given in Table 3. Twelve mothers in the intervention group and three mothers in the control group said that they fed their babies with formula for the first three days after birth. After these three days, the mothers thought that formula was not a suitable substitute for breast milk, so they started EBF and continued through the first six months. These 15 mothers who had breastfed for the first six months were also included in the study. There was a significant difference between the intervention and control groups in terms of EBF in the first six months after birth (Table 3).

**Table 3.** Frequencies of exclusive breastfeeding for six months in groups

		Intervention group (*n* = 82)	Control group (*n* = 52)	*P* value^*^
EBF(first six months after birth)	Yes	36 (43.9%)	12 (23.1%)	0.014
	No	46 (56.1%)	40 (76.9%)	

* Chi-square test.

### Initiation of supplementary food

The initiation time for supplementary food and water-fruit juice was earlier in the control group when compared to the intervention group (Table 4).

**Table 4.** Initiation time of supplementary food and juices

	Intervention group	Control group	*P* value
	Mean ± SD (week)	Mean ± SD (week)	
Supplementary food	18.5 ± 8	15 ± 9.5	0.031
Water/fruit juice	17.5 ± 8.2	11.8 ± 9.3	0.000

The most common reasons for early weaning perceived by mothers were “my breast milk is insufficient,” “the baby is hungry,” and “the provision of formula food to the babies at the hospital in the early hours following birth.”

### Difference in breastfeeding frequency between intervention groups 1 and 2

The intervention groups 1 and 2 were significantly different in terms of frequency of exclusive breastfeeding (EBF) when compared with the control group (respectively, *P* = 0.003, *P* = 0.00). But when compared one to the other, there was no significant difference between groups 1 and 2.

### Feedbacks from the intervention group at the end of training

All 82 mothers who attended the training responded positively to some questions that “Have you received satisfactory answers to the questions in your mind?”, “Was the training understandable?”, “Was the location of the training appropriate?”.

The mothers evaluated the breastfeeding training according to a five-point rating scale ranging from 0 (very bad) to 5 (very good). Eighty mothers rated the training as very good (95.2%), three mothers as good (3.6%), and one mother as acceptable (1.6%) (Table 5).

**Table 5.** Feedbacks from the intervention group at the end of training

	Yes	No
Questions	*n* (%)	*n* (%)
Did you learn something new with this training?	80 (97.5)	2 (2.5)
Did your training answer your needs?	81 (98.7)	1(1.3)
Have you been actively involved in training during training?	80 (97.5)	2 (2.5)
Has adequate practice been made in the training program?	80 (97.5)	2 (2.5)
Were there any issues that were missing in the training program?	12 (14.7)	70 (85.3)
Was the training time sufficient?	78 (95.1)	4 (4.9)

## Discussion

The WHO recommends EBF during the first six months of life and to continue up to two years of age. Even supported worldwide, the EBF rates still remain low in developing and even in the developed countries. Several interventional studies using different methods have been conducted to find out an effective way to support the mothers to start and continue breastfeeding. Timing of the education is found to be an important factor in order to create a positive effect. Educational interventions which are conducted in the perinatal period, that is, just before and after the delivery, seem to be more effective (Lumbiganon *et al*., 2012).

Since the rates of EBF in our country are still low, there is a need for similar studies including interventions adaptable to the characteristics of our population. In this study, the interactive group education and counseling in the prenatal period and professional support for correct breastfeeding provided to each participant during postnatal follow-up visits were used as intervention methods and their effects were investigated. In our study, EBF frequency was significantly higher in the intervention group compared to the control group.

The study was carried out in baby-friendly FHC. In Turkey, 95% of FHC are baby-friendly. In their recent study, Çaylan *et al.* suggested that baby-friendly health facilities are appropriate for breastfeeding promotion in Turkey (Çaylan, 2019). The results of our study support their suggestion.

DHS is a large-scale study repeated every five years and is a reliable reference for health parameters especially in reproductive health. In TDHS 2018, frequency of EBF in the fifth month decreased by 14.4% compared to the TDHS 2008 study results which were 30.1 versus 40.7% for the fifth month. In our study, the rates of EBF were 43% in the intervention group and 23% in the control group and at the end of the sixth month. The breastfeeding rates in the intervention group were higher than that the control group and of the two consecutive TDHS as well. This result can be commented as a proof for the effectiveness of the methodology of this study.

Onbasi *et al*. reported that the frequency of EBF for the sixth month was 67.8%, whereas the frequency of EBF was lower in our study. The study of Onbasi *et al*. had been conducted in Edirne, the city located in the region that has the second highest incidence of breastfeeding in Turkey according to TDHS 2018. In the mentioned study, group education had been given once to the patients who had admitted to the hospital for antenatal care and then the result of breastfeeding training was evaluated in the sixth month by using a face-to-face questionnaire. The data of TDHS 2018, compared to that of the TDHS 2008, show a lower frequency of EBF. The low prevalence of EBF in our study may reflect the general decline in the country. Nonetheless, the majority of the mothers in the Onbasi *et al*. intervention group were high school and university graduates, whereas the education level of the participants was lower in our study. The difference between the two results may be related to the difference of timing of the outcome measurement. In Onbasi *et al*.’s study, the knowledge levels of the mothers had been evaluated at the sixth month, whereas this evaluation was performed at the pre-training and post-training period in our research (Onbasi *et al*., 2011). Also, the evidence suggests that the effectiveness of prenatal breastfeeding education lasts till the fourth to sixth week maximum the third month after delivery (Imdad *et al*., 2011; Wong *et al*., 2015).

A systematic review reported that the interventions on breastfeeding increased the frequency of EBF. In this review, it was concluded that group and individual counseling together was superior to counseling provided only in groups or individually (Haroon *et al*., 2013). The results of a study conducted in the Department of Obstetrics and Gynecology, Haydarpaşa Numune Teaching Hospital (in Istanbul) support our results. In this study, authors concluded that prenatal small group sessions combined with individual problem-oriented support after the delivery increased EBF rates for the six months compared to only antenatal education (Vural and Vural, 2017). As a result, either individual breastfeeding support or group training plays an important role in increasing EBF frequency. These interventions may not protect their effectivity till the sixth month if they are only applied in the antenatal period. The duration of this positive effect may be extended if antenatal training is supported by interventions during the postnatal period.

### Conclusions and limitations

As this study was conducted on a voluntary basis, the participants of the intervention group might be more willing to breastfeed their babies. A second limitation was the low number of participants in groups, especially in the intervention group which was divided into two subgroups based on the methodology. The number of participants was lower than expected, as the 23% of the mothers started supplementary food in the second month; the participants’ number at the fourth month follow-up was lower for final analysis. Therefore, the number of participants in the intervention group decreased when compared to the control group and this had reduced the power of the study.

In conclusion, our study aimed to determine the effect of an antenatal interactive group-based educational model and follow-up on early weaning. The group training and postnatal reminders were found to be more effective than the individual training provided at the baby-friendly health institutions in terms of the effectiveness on increasing the frequency of EBF.
